# Tbet-positive regulatory T cells accumulate in oropharyngeal cancers with ongoing tumor-specific type 1 T cell responses

**DOI:** 10.1186/s40425-019-0497-0

**Published:** 2019-01-18

**Authors:** S. J. Santegoets, C. L. Duurland, E. S. Jordanova, J. J. van Ham, I. Ehsan, S. L. van Egmond, M. J. P. Welters, S. H. van der Burg

**Affiliations:** 1 Departments of Medical Oncology, Leiden University Medical Center, Albinusdreef 2, 2333 ZA Leiden, the Netherlands; 2 Department of Obstetrics and Gynecology, Center for Gynecological Oncology Amsterdam (CGOA) Amsterdam UMC, Vrije Universiteit Amsterdam, Amsterdam, the Netherlands; 3 Departments of Otorhinolaryngology and Head and Neck Surgery, Leiden University Medical Center, Leiden, the Netherlands

**Keywords:** Tumor microenvironment, Type 1 immunity, HPV, Head and neck cancer, Foxp3

## Abstract

Regulatory T cells (Tregs) may comprise different subsets allowing them to efficiently suppress different types of effector T cells. In this study, we show that high numbers of both conventional and Tbet co-expressing Foxp3^hi^ Tregs accumulate in human papilloma virus (HPV)-driven oropharyngeal squamous cell carcinoma (OPSCC). The infiltration of Tbet+ Foxp3+ Tregs was strongly correlated with a concomitant tumor-specific and conventional type 1-oriented intratumoral T cell infiltrate. Both conventional CD4+CD25+CD127–Foxp3^hi^ Tregs and their Tbet^hi^ counterparts exhibited an activated phenotype, co-expressed high levels of CTLA4 and Helios and exhibited a maximally demethylated Foxp3 gene locus TSDR, indicating their full capacity to impede a type 1 effector T cell response. Interestingly, while the prognostic value of conventional Tregs was neutral, a high intratumoral frequency of Tbet+ Tregs was associated with prolonged disease-specific survival, most likely because their presence reflected high numbers of effector T cells. The presence of these Tbet+ Tregs may in part explain why a dense type 1-oriented immune infiltrate in OPSCC is not enough to fully control tumor growth.

**Electronic supplementary material:**

The online version of this article (10.1186/s40425-019-0497-0) contains supplementary material, which is available to authorized users.

## Introduction

Foxp3+ regulatory T cells (Tregs) are pivotal in suppressing pathological and physiological immune responses [1]. In cancer, Tregs are generally considered to suppress the local tumor immune response. However, different types of impact have been reported for Tregs in patients suffering from head and neck cancer, including oropharyngeal squamous cell carcinoma (OPSCC) [2]. Some studies suggested a negative prognostic value for high frequencies of tumor-infiltrating CD4+CD25+Foxp3+ Tregs in head and neck cancer [3–5], whereas others demonstrated a non-predictive [6] or even positive prognostic role [7–10] for these cells.

Recent studies have shown that Tregs can adopt different transcriptional profiles allowing them to regulate specific types of effector T cells [11]. In mice, Tregs express the transcription factor STAT3 to restrain Th17 responses [12], IRF4 to suppress Th2 responses [13] and Tbet to suppress Th1 responses in persistent infections or autoimmunity [14, 15]. Foxp3+Tbet+ Tregs are phenotypically stable [15] and regulate Th1 inflammatory sites in vivo, thereby counterbalancing unwanted tissue destruction and immunopathology [14].

Since many head and neck cancers can be infiltrated with type 1-oriented T cells [16], we hypothesized that this would also attract type 1-oriented Tregs. Therefore, the presence and potential impact of conventional and Tbet-expressing Foxp3+ Tregs was analyzed in the context of a prospective study on the immune response in HPV-induced OPSCC [16] by three-color immunofluorescence staining and 12-parameter flow cytometry analysis. We show that high numbers of activated bona fide Foxp3+Tbet+ Tregs accumulate in OPSCC when a concomitant HPV-specific and a type 1-oriented T cell infiltrate is present.

## Materials and methods

The authors acknowledge the reporting of Minimal Information About T-cell Assays (MIATA).

### Patients

The 50 OPSCC patients were part of an observational study (P07–112) investigating immunity in patients with head and neck cancer [16]. The 19 patients with cervical carcinoma (CxCa) that were included in this study took part in the CIRCLE 2 study (P08–197) investigating cellular immunity against anogenital lesions [17]. All patients were included after signing informed consent. The study was conducted in accordance with the Declaration of Helsinki and approved by the local medical ethical committee of the Leiden University Medical Center (LUMC) and in agreement with the Dutch law. Human papillomavirus (HPV) typing and p16^ink4a^ IHC staining was performed on formalin-fixed paraffin-embedded (FFPE) tumor sections as described [10]. An overview of patient characteristics is given in Additional file 1: Table S1.

### Immunofluorescence

Characterization of lymphocytic infiltration was carried out with triple immunofluorescent staining in 41 OPSCC as described previously [10] using anti-CD8 (mouse IgG2b, clone 4B11; Novocastra, 1:400), anti-Tbet (rabbit polyclonal, clone H210; Santa Cruz 1:400) and anti-Foxp3 (mouse IgG1, clone 236A/E7; Abcam, 1:200), goat-anti-mouse IgG2b Alexa 647, goat-anti-rabbit Alexa 546 and goat-anti-mouse IgG1 Alexa 488 (all from Molecular probes; 1:200). Based on the morphology of cancer cell nests and autofluorescence of keratinocytes the immune cells per mm^2^ were manually counted as intraepithelial or stromal using the LSM 5 Image Examiner software (average of five images at a 250× magnification).

### Blood and tumor cell isolation and culturing

Venous blood samples were drawn prior to surgery, and peripheral blood mononuclear cells (PBMC) were isolated as described previously [16]. OPSCC tumors were obtained and handled as described previously [16, 17]. For two OPSCC tumors (H202, H206), the approach to prepare single cell suspensions was adjusted. The DNAse I/collagenase D enzymes were replaced by 0.38 mg/ml of the commercially available Liberase enzymes (Liberase TL, research grade, Roche), the incubation period was reduced to 15 min, and the GentleMACS dissociator was no longer used.

### Tumor-specific T cell reactivity analysis

Cultured tumor infiltrating lymphocytes (TILs) were analyzed for the presence of HPV16-specific T cells using a 5-day [3H]-thymidine-based proliferation assay and antigen-specific cytokine production assay as described previously [16, 18]. To this end, T cell responses against autologous HPV16 E6/E7 synthetic long peptide (SLP; 22-mers with 14 amino acids overlap) loaded monocytes were tested in triplicate. PHA (0.5 μg/ml; HA16 Remel; ThermoFischer Scientific) was taken along as positive control, while unloaded monocytes served as negative control. At day 4 supernatant (50 μl/well) was harvested to determine cytokine production. During the last 16 h of culture, 0.5 μCi/well of [3H]thymidine was added to measure proliferation. The stimulation index was calculated as the average of test wells divided by the average of the medium control wells. A positive response was defined as a stimulation index of at least 3.

Antigen-specific cytokine production was determined by cytometric bead array (CBA, Th1/Th2 kit, BD Bioscience, Breda, the Netherlands) according to the manufacturer’s instructions. The cutoff value for cytokine production was 20 pg/ml, except for IFNγ for which it was 100 pg/ml. Positive cytokine production was defined as at least twice above that of the unstimulated cells. An example of such an HPV16-specific T cell reactivity test is depicted in Additional file 2: Figure S1.

### Flow cytometry and cell sorting

Cryopreserved PBMC and/or single cell tumor samples were thawed and Treg subsets were assessed by flow cytometry as described before [19]. Antibodies and kits used were V500-labeled CD3 (clone UCHT1, BD Biosciences), AF700- or PE-Cy7-labeled CD4 (clone RPA-T4 and SK3 respectively, BD Biosciences), PE-CY7- or BV421-labeled CD25 (clone 2A3, BD Biosciences), BV650-labeled CD127 (clone A019D5, Biolegend), APC-H7-labeled CD45RA (clone HI100, BD Biosciences), PerCP-Cy5.5-labeled CD8 (clone SK1, BD Biosciences), PE-CF594- or Alexa Fluor 647-labeled Foxp3 (clone 259D/C7, BD Biosciences), BV421-labeled CTLA-4 (clone BNI3, BD Biosciences), FITC-labeled Ki67 (clone 20Raj1; eBiosciences), APC-labeled Helios (clone 22F6, Biolegend), PE-labeled Tbet (clone ebio4B10; eBiosciences), FITC-labeled CD14 (clone M5E2; BD Biosciences), LIVE-DEAD® Fixable yellow or near-IR dead cell stain kit (ThermoFisher Scientific), and the BD Transcription Factor Buffer set. Acquisition of cells was performed on a BD LSR Fortessa and flow cytometric sorting was done using a BD FACS Aria II. Data was analysed using DIVA software (version 8.02; BD Biosciences).

### DNA isolation, bisulfite conversion and FOXP3 TSDR methylation assay

Sorted Foxp3– Tconv, Foxp3+Tbet+ Treg and Foxp3+Tbet– Treg cells (ranging between 240 and 35,000 cells) were digested with Proteinase K for 4 h at 50 °C to obtain genomic DNA, followed by Bisulfite Conversion using the EZ DNA Methylation-Direct Kit (Zymo Research) according to the manufacturer. The Foxp3 Treg specific demethylation region (TSDR) was amplified (Foxp3 TSDR Forward 5′-TGG GGG TAG AGG ATT TAG AGG-3′, Foxp3 TSDR Reverse 5’-TCA CCC CAC CTA AAC CAA A C- 3′ (adapted from [20]) using platinum high-fidelity Taq (Invitrogen, Thermo Fisher Scientific). PCR products were cloned using a TOPO-TA cloning kit (Invitrogen, Thermo Fisher Scientific) [21]. Plasmid DNA was purified using the NucleoSpin Plasmid kit (Macherey-Nagel). Seventeen to twenty-four clones per population per patient were sequenced using M13 primers (M13 Forward 5′-GTA AAA CGA CGG CCA G-3′, M13 Reverse 5’-CAG GAA ACA GCT ATG AC-3′) and aligned to an imputed TSDR sequence [21] using SnapGene software (version 4.1.9, GSL Biotech LLC, snapgene.com). Methylated CpGs were identified by the presence of a C nucleotide at the CpG position whereas demethylated CpGs were identified by a T nucleotide. The percentage of clones showing methylation at each individual CpG as well as the total average of all CpGs per population per patient was determined.

### Statistical analysis

Non-parametric Wilcoxon signed-rank or Mann–Whitney tests and parametric paired or unpaired *t* tests were performed when appropriate. Correlation analysis were done the using Pearson’s correlation test. For survival analysis, patients were grouped into two groups according to the median (i.e., grouped into below or above the median of the total group for each parameter), after which survival was tested using Kaplan–Meier method, and statistical significance of the survival distribution was analyzed by log-rank testing. All statistical tests were performed at the 0.05 significance level, and differences were considered significant when *p* < 0.05, as indicated with an asterisk (*p < 0.05, **p < 0.01, *** *p* < 0.001 and **** *p* < 0.0001). Statistical analyses were performed using GraphPad Prism 7.1 (San Diego, USA).

## Results

### High numbers of Foxp3+Tbet+ Treg cells are found in the OPSCC microenvironment

**Fig. 1:**
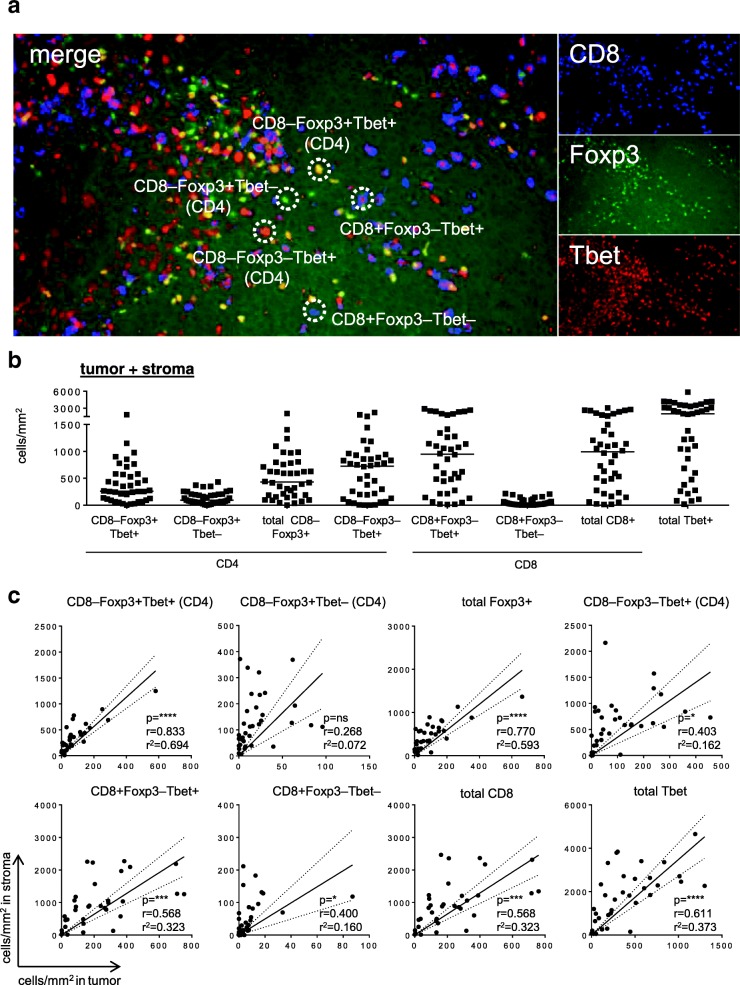
The TME of OPSCC tumors is highly infiltrated with Th1-like T cells. Paraffin-embedded OPSCC tissue of 41 patients was analyzed by triple immunofluorescent confocal microscopy analysis with antibodies directed against CD8 (blue), Foxp3 (green) and Tbet (red). **a** Example of a triple immunofluorescent staining is given for a representative OPSCC sample. Triple staining results in: CD8+Foxp3–Tbet– (blue), CD8+Foxp3–Tbet+ (blue with red nucleus), CD8–Foxp3+Tbet– (CD4; green nucleus), CD8–Foxp3+Tbet+ (CD4; yellow nucleus) and CD8–Foxp3–Tbet+ (CD4; red nucleus) cells. **b** Scatter plot depicting (from left to right) CD8–Foxp3+Tbet+, CD8–Foxp3+Tbet–, total CD8–Foxp3+, CD8–Foxp3–Tbet+ (all CD4), CD8+Foxp3–Tbet+, CD8+Foxp3–Tbet–, total CD8+ (all CD8) and total Tbet+ (CD4 and CD8) T cell infiltrates/mm2 for each patient. Cells in the epithelium (tumor) and stroma were manually counted and represented as the number of cells per mm^2^ for each slide (average of five images at 250× magnification). **c** Scatter plots and correlation analysis between the number of cells/mm2 in the stroma (Y-axis) and the tumor (X-axis) for CD8–Foxp3+Tbet+, CD8–Foxp3+Tbet–, total CD8–Foxp3+, CD8–Foxp3–Tbet+ (all CD4; top), and CD8+Foxp3–Tbet+, CD8+Foxp3–Tbet–, total CD8+ (all CD8) and total Tbet+ (CD4 and CD8; bottom) T cell infiltrates. Pearson’s correlation with the correlation coefficient (r), the coefficient of determination (r^2^) and *p* value is depicted for each correlation analysis. * *p* < 0.05, ** *p* < 0.01, *** *p* < 0.001 and, **** *p* < 0.0001. The dotted lines represent the 95% confidence interval

To assess the number of conventional and Tbet-expressing Foxp3+ Tregs in cancer patients, we analyzed 41 primary OPSCC tumors by three-color immunofluorescent confocal microscopy. An example of the CD8, Foxp3 and Tbet immunofluorescent staining is depicted in Fig. 1a. As shown in Fig. 1a and b, OPSCC were highly infiltrated with CD8+Foxp3–Tbet+ (mean 1028 cells/mm2; range 12.1–2288) and CD8–Foxp3–Tbet+ (most likely CD4; mean 639 cells/mm2; range 0–2216) T cells. In addition, substantial numbers of co-infiltrating CD8–Foxp3+Tbet– (i.e. CD4+) conventional Treg cells (mean 138.1 cells/mm2; range 0.6–430.2) and CD8–Foxp3+Tbet+ Tregs (mean 392.9 cells/mm2; range 1.2–1829) were detected. All cell types were found both in the tumor epithelium and the stroma (Fig. 1a and Additional file 2: Figure S2), albeit that the stroma was more densely infiltrated (*p* < 0.0001 for all subsets). Furthermore, the numbers of stromal and intraepithelial CD8–Foxp3–Tbet+ (CD4), CD8+Foxp3–Tbet+ and in particular CD8–Foxp3+Tbet+ Treg cells were significantly correlated. This was not the case for the conventional Tregs, which were more often found in the tumor stroma (Fig. 1c).

### The numbers of tumor-infiltrating CD8–Foxp3+Tbet+ Treg cells correlate directly to a strong infiltration with type 1-oriented CD4+ and CD8+ effector T cells

**Fig. 2:**
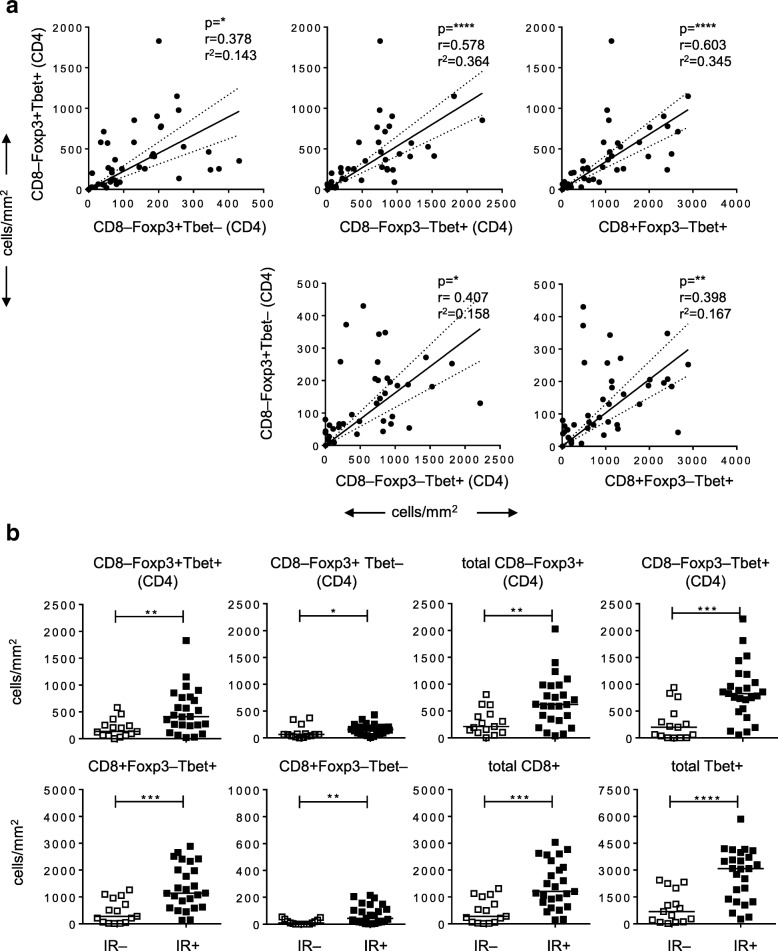
Foxp3+Tbet+ Tregs are attracted to Th1-oriented OPSCC tumors. **a** Scatter plots with correlation analysis between the number of CD8–Foxp3+Tbet+ cells and CD8–Foxp3+Tbet–, CD8–Foxp3–Tbet+ (CD4) and CD8+Foxp3–Tbet– cells (top) and between the number of CD8–Foxp3+Tbet– cells and CD8–Foxp3–Tbet+ (CD4) and CD8+Foxp3–Tbet cells (bottom). Pearson’s correlation with the correlation coefficient (r), the coefficient of determination (r^2^) and *p*-value is depicted for each correlation analysis. The dotted lines represent the 95% confidence interval. **b** Scatter plots depicting (from left to right) CD8–Foxp3+Tbet+, CD8–Foxp3+Tbet–, total CD8–Foxp3+, CD8–Foxp3–Tbet+ (all CD4; top), CD8+Foxp3–Tbet+, CD8+Foxp3–Tbet–, total CD8+ and total Tbet+ (CD4 and CD8; bottom) T cell infiltrates/mm2 in 15 immune response-negative (IR–; open squares) and 26 IR+ (closed squares) OPSCC patients. Data for CD8–Foxp3–Tbet+ and CD8+Foxp3–Tbet+ T cells have been described before [16]. * *p* < 0.05, ** *p* < 0.01, *** *p* < 0.001 and, **** *p* < 0.0001

Next, we assessed if there was a correlation between the number of Foxp3+Tbet+ Tregs and the presence of a type 1-oriented tumor immune infiltrate (Fig. 2a). A positive correlation was found between the numbers of tumor-infiltrating CD8–Foxp3+Tbet+ Tregs, CD8–Foxp3–Tbet+ (CD4) T cells and CD8 + Foxp3–Tbet+ T cells, supporting the notion that CD8–Foxp3+Tbet+ Tregs accumulate at similar sites as type 1-oriented (Foxp3-) effector T cells. Indeed, only a weak correlation between the density of Foxp3+Tbet– Tregs and these tumor-infiltrating Tbet+ T cell subtypes was observed.

The presence of intratumoral Tbet+ T cells suggests, but does not demonstrate, the presence of tumor-specific IFNγ-producing T cells that can stimulate the accumulation of CD8–Foxp3+Tbet+ Tregs. Hence, we analyzed their relation with the detection of HPV16 E6 and E7 oncoprotein-specific IFNγ and TNFα producing CD4+ and CD8+ T cells in these tumors [16]. The OPSCC patients were divided into two groups based on the presence (+) or absence (−) of an HPV16-specific T cell immune response (IR) among the TIL cultured from these tumors (Additional file 1: Table S1; Additional file 2: Figure S1). This revealed that OPSCC containing HPV-specific T cells also displayed higher numbers of all the T cell populations, and in particular also higher numbers of CD8–Foxp3+Tbet+ Tregs (Fig. 2b). Notably, the correlation between the number of CD8–Foxp3+Tbet+ Tregs and CD8–Foxp3–Tbet+ (CD4) T cells or CD8+Foxp3–Tbet+ T cells is retained in both the IR- and IR+ groups, however, in IR- patients the number of these 3 cell types is lower than in the IR+ group (Additional file 2: Figure S3).

### Tbet– And Tbet+ CD4+CD25+CD127–Foxp3^hi^ Tregs are bonafide activated Tregs

**Fig. 3:**
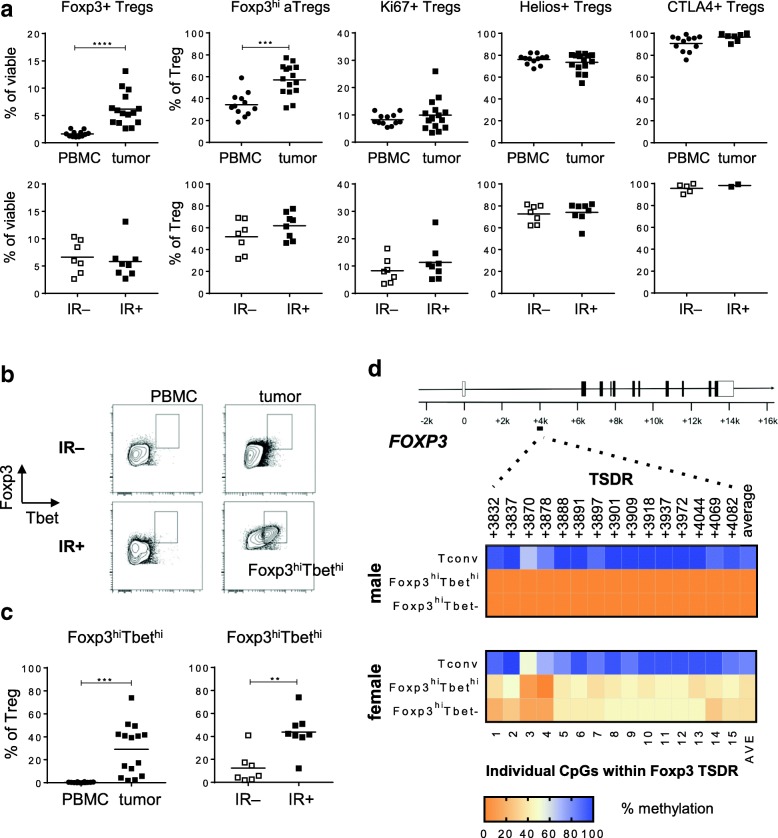
Tumor infiltrating Tbet– and Tbet^hi^ CD4+CD25+CD127-Foxp3^hi^ cells are bona fide activated Tregs. Freshly dissociated OPSCC tumor tissue was analyzed by 12-parameter flow cytometry analysis with antibodies directed against CD3, CD4, CD8, CD25, CD127, Foxp3, CD45RA, Ki67, Helios, CTLA-4 and Tbet. **a** Scatter plots displaying the percentage of CD4+CD25+CD127–Foxp3+ Tregs as percentage of viable cells, and the percentage of Foxp3^hi^CD45RA–, Ki67+, Helios+ and CTLA4+ Tregs of percentage CD4+CD25+CD127–Foxp3+ Treg within PBMC (closed circles) and tumor (closed squares; top panel) samples and within immune response-negative (IR-; open squares) and IR+ (closed squares; bottom panel) tumors of 15 OPSCC patients. **b** Representative examples of the Foxp3 / Tbet staining within PBMC (left) and tumor (right) of a IR– (top) and IR+ (bottom) OPSCC patient are shown. Cells depicted were first gated for viable and single cells, and further gated for expression of CD3, CD4, CD25, absence of CD127 and expression of Foxp3. **c** Scatter plots displaying the proportion of Foxp3^hi^Tbet^hi^ Tregs as percentage of viable cells (left), and as percentage of CD4+CD25+CD127–Foxp3+ Tregs within OPSCC PBMC (closed circles) and tumor (closed squares; left panel) and within IR– (open squares) and IR+ (closed squares; right panel) OPSCC tumors. * *p* < 0.05, ** *p* < 0.01, *** *p* < 0.001 and, **** *p* < 0.0001. **d** Heat map plots displaying the methylation percentages of the individual CpG sites within the Foxp3 Treg cell-specific demethylated region (TSDR) and the average (AVE) of the 15 CpG sites for CD4+CD14–CD25–CD127+Foxp3– conventional cells (Tconv), CD4+CD14–CD25+CD127–Foxp3^hi^Tbet^hi^ and Foxp3^hi^Tbet– cells from a male (top) and female (bottom) donor. Populations were isolated from dissociated OPSCC tumors following FACS sorting on a BD FACS Aria II. Percentage methylation is depicted in color-code as depicted in the legend. Treg cells are partially demethylated in the female donor due to Foxp3 methylation on the inactive X-chromosome

To further characterize the conventional and Tbet-expressing Tregs, we used our consensus Treg marker set and robust gating strategy for flow cytometry analysis of Tregs [19] for 15 freshly dissociated OPSCC and matching PBMC samples. An example of our gating strategy is given in Additional file 2: Figure S4. Much higher frequencies of CD4+CD25+CD127–Foxp3+ Tregs were detected in OPSCC tissue compared to PBMC (Fig. 3a), indicating their tumor-specific accumulation. Furthermore, the majority of the intratumoral Treg cells exhibited a Foxp3^hi^CD45RA– activated Treg (Foxp3^hi^ aTreg) profile, and expressed high levels of the Treg-associated markers CTLA-4 and Helios. Notably, the Tbet^hi^Foxp3^hi^ Tregs expressed higher levels of helios, CTLA-4 and Ki67 than the Tbet– Foxp3+ Tregs (Additional file 2: Figure S5), suggesting that they have been stimulated and activated in the tumor microenvironment [22]. Importantly, the levels of total CD4+CD25+CD127–Foxp3+ Tregs within the viable tumor-infiltrating lymphocyte fraction, as well as the proportion of Foxp3^hi^ aTregs, and Ki67+, Helios+ and CTLA4+ conventional CD4+CD25+CD127–Foxp3+ Tregs, were not related to the detection of a concomitant intratumoral HPV16-specific type 1 T cell response (Fig. 3a). In contrast, Tbet^hi^ Tregs were specifically enriched in the tumors harboring such a HPV-specific T cell response (Fig. 3b and c). In addition, the levels of tumor-infiltrating Foxp3^hi^Tbet^hi^ Tregs strongly correlated with the levels of infiltrating CD4+Tbet+ and CD8+Tbet+ cells (Additional file 2: Figure S6). To analyze whether the levels of tumor-infiltrating Foxp3^hi^Tbet^hi^ Tregs could be linked to a more activated tumor immune profile, we analyzed the activation status of the tumor-infiltrating CD4+ and CD8+ T cells in a cohort of 13 previously studied OPSCC tumors [18], of which 8 were also included here. Based on this limited set of samples, IR+ OPSCC tumors contained higher percentages of highly activated PD1^hi^HLA-DR^hi^ CD4 and CD8 T cells, and their levels correlated with the number of tumor-infiltrating Tbet^hi^Tregs (Additional file 2: Figure S6 b-e).

To assess if the detected Foxp3^hi^Tbet^hi^ T cells are truly Tregs, we sorted Foxp3^hi^Tbet^hi^ and Foxp3^hi^Tbet– Treg populations by flowcytometry from HPV16+ OPSCC, of which we knew that they contained Tbet^hi^ T cells and HPV16-specific T cells as well as for which freshly digested tumor material was still available (Additional file 2: Figure S7). Previously it has been shown that the TSDR, a conserved region in intron 1 of *FOXP3*, is strictly hypomethylated in Tregs whereas this region is hypermethylated in conventional T cells and T cell clones, even after activation [20, 23] and, therefore, the TSDR methylation status functions as a true Treg specific parameter [24]. Indeed, epigenetic analysis revealed that Foxp3– conventional T cells (Tconv) exhibited maximal TDSR methylation whereas this region was strongly hypomethylated not only in the conventional Foxp3^hi^Tbet– cells (> 95% for male and 50% for female donor due to Foxp3 methylation on the inactive X-chromosome for all 15 different CpG islands in this region) but also in the Foxp3^hi^Tbet^hi^ cells (Fig. 3d), indicating that Foxp3^hi^Tbet^hi^ and Foxp3^hi^Tbet– Tregs present in these tumors indeed are bona fide Tregs.

**Fig. 4:**
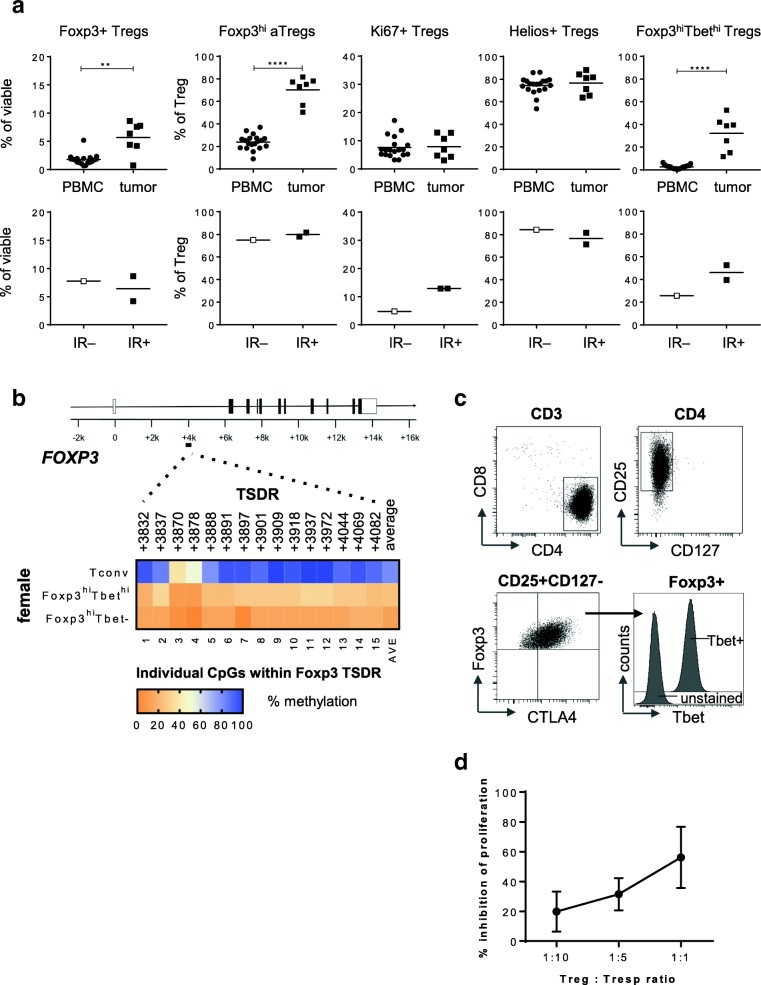
Foxp3^hi^Tbet^hi^ Tregs accumulate in CxCa tumors with an ongoing type 1 immune response. Freshly dissociated CxCa tumor tissue was analyzed by 12-parameter flow cytometry analysis with antibodies directed against CD3, CD4, CD8, CD25, CD127, Foxp3, CD45RA, Ki67, Helios, CTLA-4 and Tbet. **a** Scatter plots displaying the percentage of CD4+CD25+CD127–Foxp3+ Tregs as percentage of viable cells, and the percentage of Foxp3^hi^CD45RA– activated Tregs (Foxp3^hi^ aTregs), and Ki67+, Helios+ and Foxp3^hi^Tbet^hi^ Tregs of percentage CD4+CD25+CD127–Foxp3+ Treg within CxCa PBMC (closed circles) and tumor (closed squares; top panel) and within immune response-negative (IR–; open squares) and IR+ (closed squares; bottom panel) CxCa tumors. * *p* < 0.05, ** *p* < 0.01, *** *p* < 0.001 and, **** *p* < 0.0001. **b** Heat map plots displaying the methylation percentages of the individual CpG sites within the Foxp3 Treg cell-specific demethylated region (TSDR) and the average of the 15 CpG sites for Tconv, Foxp3^hi^Tbet^hi^ and Foxp3^hi^Tbet– cells from a female CxCa donor. Percentage methylation is depicted in de color-code as depicted in the legend. Treg cells are partially demethylated in the female donor due to X-inactivation**. c** FACS plots depicting the CD25, CD127, Foxp3, CTLA4 and Tbet expression in the CD4+Foxp3^hi^ Treg clone C148.31, which was derived from an HPV16+ cervical patient with an ongoing local type 1 immune response [26]. In the right bottom graph, an histogram overlay of Tbet over unstained control is depicted. **d** Graph showing percentage inhibition of responder T cell (Tresp) proliferation by the C148.31 CD4+Foxp3^hi^ Treg clone at 1:10, 1:5 and 1:1 Treg: Tresp ratios. Mean and SD of three independent tests

In order to understand if such Foxp3^hi^Tbet^hi^ Tregs also accumulate in other inflamed tumors infiltrated by Tbet+ T cells such as HPV-driven cervical carcinoma (CxCa) [25], seven freshly dissociated CxCa tumor samples were analyzed. Again, high frequencies of Foxp3^hi^Tbet^hi^ Tregs specifically accumulated in CxCa tissue and these Tregs also displayed a hypomethylated TDSR locus. Although only three out of seven CxCa tumors were HPV16-driven, a similar association between the higher levels of Foxp3^hi^Tbet^hi^ Tregs and the detection of an HPV16-specific immune response was found (Fig. 4).

Thus, Foxp3^hi^Tbet^hi^ cells accumulate specifically in tumors with an ongoing type 1 immune response and based on the hypomethylation of all 15 CpG islands in the TDSR region of the Foxp3 intron 1 in these T cells sorted from 3 different patients, are true regulatory T cells.

Last but not least, we analyzed the expression of Tbet in CD4+Foxp3^hi^ regulatory T cell clone 148.31, isolated from an HPV16+ cervical cancer patient with an ongoing local type 1 immune response, and for which we have shown its suppressive capacity in several different in vitro assays [26]. This clone not only was suppressive but in addition to Foxp3 also expressed the transcription factor Tbet (Fig. 4c, d).

### High numbers of tumor-infiltrating Foxp3^hi^Tbet^hi^ Tregs, but not conventional Tregs, correlate with improved survival

**Fig. 5:**
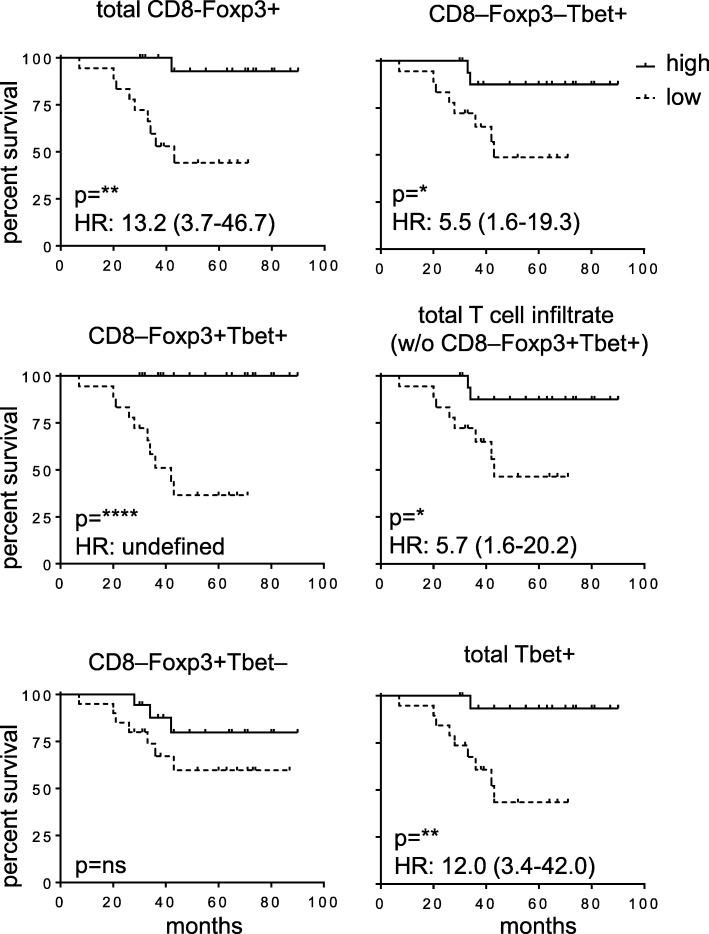
High frequencies of tumor-infiltrating Foxp3+ Tbet+ Tregs correlate with survival in OPSCC patients. Kaplan-Meier survival curves showing the outcome of 38 tested HPV16-positive OPSCC patients with high (above the median) and low (below the median) numbers of total CD8–Foxp3+, CD8–Foxp3+Tbet+ and CD8–Foxp3+Tbet– (top to bottom; left), and CD8–Foxp3–Tbet+, total T cell infiltrate (without CD8–Foxp3+Tbet+ cells) and total Tbet+ T cells/mm2 (top to bottom; right). T cell subsets were determined by IHC staining. Statistical significance of the survival distribution was analyzed by log-rank testing and differences were considered significant when *p* < 0.05, as indicated with an asterisk (*p < 0.05, **p < 0.01, *** *p* < 0.001 and **** *p* < 0.0001)

Now that it was clear that at Tbet^hi^ and Tbet- Tregs infiltrated OPSCC, the prognostic value of both types of Tregs in our OPSCC patient cohort was analyzed. This revealed that the total population of CD8–Foxp3+ Tregs was significantly associated with improved survival (Fig. 5). However, analysis of the individual prognostic impact of either conventional CD8–Foxp3+Tbet– Tregs or CD8–Foxp3+Tbet+ Tregs, revealed that infiltration with conventional Tregs had no significant impact on survival whereas high numbers of CD8–Foxp3+Tbet+ Tregs were highly correlated with improved survival after (chemo)radiation therapy (Fig. 5). Thus, depending on the subset of Tregs analyzed, either a neutral or a positive impact on prognosis was found.

## Discussion

In mice, Foxp3+Tbet+ Tregs were shown to play a role in suppressing Th1-like effector T cell responses [14, 15]. We previously showed that a dense type 1-oriented immune infiltrate is present in a large majority of patients with HPV16-driven oropharyngeal and cervical cancers [16, 25] and wondered if these tumors are infiltrated with a similar type of Treg. Not only did we detect high numbers of CD4+ T cells co-expressing both the transcription factors Foxp3 and Tbet in these HPV16-driven tumors but we also found that their numbers are directly related to the number of tumor-infiltrating type 1-oriented (Foxp3-) effector T cells. Due to the inability to isolate viable Foxp3+Tbet+ and Foxp3+Tbet– Tregs by flowcytometry using antibodies against Foxp3 and Tbet, these subtypes of Tregs could not be tested in a functional T cell suppression assay. Therefore, we used the well accepted surrogate for functionality by assessing the methylation status of the FoxP3 gene locus TSDR. Hypomethylation of this locus is generally considered the gold standard for identifying functional Tregs [20, 23]. This method is widely used to distinguish true Tregs from other T cell populations which transiently upregulate FOXP3 after their activation [24, 27] and it showed that the CD4+Foxp3^hi^Tbet^hi^ cells found in HPV-driven cancers represent bona fide Tregs. The fact that the in vitro proven suppressive CD4+Foxp3^hi^ Treg clone 148.31, isolated from a patient with an ongoing HPV-specific type 1 T cell response [26], also co-expresses Tbet corroborates our findings.

The detection of conventional and Tbet+ Treg populations in OPSCC and CxCa confirmed studies reporting the presence of CD4+Foxp3+ T cells co-expressing Tbet in chronic hepatitis C driven liver cancer, oral squamous cell carcinoma and ovarian cancer [28–30]. Importantly, we found that Foxp3^hi^Tbet^hi^ Tregs specifically dominated in those cancers where an ongoing HPV-specific type 1 T cell response could be detected. In mice, it seems that Tbet+ Tregs differentiate de novo from their Tbet– counterparts under situations that drive type 1 immunity. Elimination of these Tbet+ Tregs results in severe Th1 autoimmunity [15] indicating that their purpose is to keep a type 1-oriented immune response at bay to prevent immune pathology. In human tumors, they are expected to do the same. Our results on the prognostic value of the two different populations for patient survival, therefore, are a bit counterintuitive. The observations that conventional Tregs more often were found in the tumor stroma and correlated less with total effector cell infiltration may explain why they had no impact on survival. In contrast, the Tbet^hi^ Tregs do infiltrate the tumor cell nests and their number strongly correlated with that of tumor-infiltrating Tbet+ CD4+ and CD8+ effector T cells as well as with the detection of ongoing HPV-specific type 1 T cell responses. Yet unexpectedly, their infiltration has a strong favorable prognostic value for survival. It should, however, be realized that the number of Tbet+ non-regulatory T cells in these tumors is much higher and the presence of these type 1 effector T cells is associated with improved survival in OPSCC (Fig. 5 and reference [16]). Hence, it is more likely that the association between high numbers of true regulatory Tbet+ T cells, based on the hypomethylated TSDR and improved survival is a reflection of the presence and clinical impact of a strong ongoing and protective type 1 tumor-specific immune response, than that these Tbet+ Tregs would have a positive contribution to the antitumor response. This view fits with previous studies showing no [6, 7] or a positive [8, 10] prognostic effect of Foxp3+ Tregs in head and neck cancer, as here the number of co-infiltrating (activated) CD4+ and CD8+ T cells is also much higher than that of Tregs. In those situations that a negative impact of Tregs was found, the tumors were scarcely infiltrated with T cells [3, 4]. Thus, Foxp3+Tbet+ Treg cells accumulate at the OPSCC tumor sites where they probably exert a suppressive effect that is strong enough to impede full type 1 effector T cell-mediated control of the tumor (i.e. spontaneous tumor regression). However, given the observed survival benefit for patients with an HPV-specific and type 1-oriented intratumoral T cell infiltrate, their numbers or activity may be insufficient to fully suppress the ongoing clinically beneficial anti-tumor response after standard therapy.

## Additional files

Additional file 1: **Table S1.** Patient characteristics. (DOCX 22 kb)

Additional File 2: **Figure S1.** HPV16 E6/E7 specific T cell reactivity testing. **Figure S2.** Conventional (Tbet–) and Tbet+Foxp3+ Tregs can be found in tumor and stroma of OPSCC samples.** Figure S3.** The correlation between the number of CD8–Foxp3 + Tbet+ Tregs and CD8–Foxp3–Tbet+ (CD4) T cells and CD8 + Foxp3–Tbet+ T cells is retained in IR- and IR+ OPSCC tumors. **Figure S4.** Gating strategy for Treg subpopulations.** Figure S5.** TbethiFoxp3+ Tregs express higher levels of helios, CTLA4 and Ki67.** Figure S6.** The levels of tumor-infiltrating Foxp3hiTbethi Tregs correlated with the levels of infiltrating CD4 + Tbet+ and CD8 + Tbet+ cells, as well as with levels of highly activated infiltrating CD4+ and CD8+ T cells. **Figure S7.** Sorting of Tregs. (PPTX3379 kb)
