# Supplementary material for: Tbet-positive regulatory T cells accumulate in oropharyngeal cancers with ongoing tumor-specific type 1 T cell responses
Source: J Immunother Cancer. 2019 Jan 18;7:14. doi: 10.1186/s40425-019-0497-0 (PMC6339415; doi:10.1186/s40425-019-0497-0)
Supplement: Supplementary file 1 — Table S1. Patient characteristics. (DOCX 22 kb) [file 40425_2019_497_MOESM1_ESM.docx]

| ID^*^ | Sex | Age | Tumor location | pTNM stage | HPV16 status | p16 status | Immune response status^$^ |
| --- | --- | --- | --- | --- | --- | --- | --- |
| H27 | F | 50 | Tonsil | T1-N0-M0 | + | + | + |
| H30 | F | 68 | Tonsillar Fossa | T3-N0-M0 | + | + | + |
| H31 | F | 63 | Tonsil | T3-N2b-M0 | + | ‒ | ‒ |
| H35 | M | 48 | Tonsillar Fossa | T1-N2b-M0 | + | + | + |
| H46 | M | 60 | Tongue base | T3-N2c-M0 | + | + | + |
| H53 | M | 54 | Lateral wall | T2-N2b-M0 | + | + | + |
| H56 | F | 64 | Tongue base | T4a-N0-M0 | + | ‒ | ‒ |
| H59 | F | 64 | Tongue base | T3-N2c-M0 | + | + | ‒ |
| H61 | F | 65 | Tonsil | T2-N1-M0 | + | + | + |
| H62 | M | 62 | Tongue base | T4a-N2c-M0 | + | ‒ | ‒ |
| H63 | F | 61 | Tonsil | T3-N2c-M0 | + | ‒ | ‒ |
| H64 | M | 60 | Tongue base | T4a-N2b-M0 | + | ‒ | ‒ |
| H66 | M | 53 | Tongue base | T1-N2a-M0 | + | + | + |
| H67 | F | 51 | Tonsil | T2-N1-M0 | + | + | + |
| H68 | F | 65 | Tonsil | T3-N2b-M0 | + | + | + |
| H70 | M | 65 | Tongue base | T1-N1-M0 | + | + | + |
| H71 | F | 63 | Tongue base | T2-N2c-M0 | + | + | + |
| H73 | M | 67 | Tongue base | T1-N2b-M0 | + | + | + |
| H74 | F | 65 | Tonsil | T4a-N0-M0 | ‒ | ‒ | ‒ |
| H77 | M | 48 | Tonsillar Fossa | T1-N2a-M0 | + | + | + |
| H80 | M | 54 | Tonsil | T3-N0-M0 | + | + | + |
| H81 | M | 48 | Tonsillar Fossa | T2-N2b-M0 | + | + | ‒ |
| H93 | M | 57 | Tongue base | T1-N2b-M0 | + | + | + |
| H95 | M | 59 | Tongue base | T3-N1-M0 | + | ‒ | ‒ |
| H96 | F | 56 | Tongue base | T3-N2c-M0 | + | ‒ | ‒ |
| H97 | M | 70 | Posterior wall | T3-N0-M0 | + | ‒ | ‒ |
| H103 | M | 66 | Tonsil | T1-N2b-M0 | + | + | + |
| H104 | F | 78 | Tongue base | T2-N2a-M0 | + | + | + |
| H133 | M | 53 | Tonsillar Fossa | T2-N0-M0 | + | + | + |
| H136 | F | 47 | Tonsil | T2-N3-M0 | + | + | + |
| H137 | F | 73 | Tongue base | T3-N2c-M0 | + | + | ‒ |
| H138 | M | 75 | Tongue base | T2-N0-M0 | + | + | + |
| H139 | M | 57 | Tongue base | T2-N3-M0 | + | + | ‒ |
| H141 | M | 68 | Tongue base | T3-N2b-M0 | + | + | + |
| H143 | F | 75 | Lateral wall | T1-N2a-M0 | ‒ | + | ‒ |
| H145 | M | 47 | Tonsil | T1-N2b-M0 | + | + | + |
| H147 | M | 57 | Tonsil | T2-N3-M0 | + | + | ‒ |
| H148 | F | 65 | Tonsil | T2-N2b-M0 | + | + | + |
| H149 | F | 48 | Tonsil | T2-N2a-M0 | + | + | ‒ |
| H150 | M | 60 | Tonsil | T2-N2b-M0 | + | + | ‒ |
| H151 | M | 72 | Vallecula | T3-N0-M0 | ‒ | ‒ | ‒ |
| H158 | M | 70 | Tongue base | T2-N2b-M0 | + | + | + |
| H159 | F | 68 | Tonsil | T4a-N2b-M0 | + | + | + |
| H161 | F | 66 | Tongue base | T2-N2b-M0 | + | + | + |
| H176 | M | 53 | Tongue base | T3-N0-M0 | + | + | + |
| H180 | M | 59 | Tonsil | T2-N2a-M0 | + | + | + |
| H182 | M | 66 | Tongue base | T4-N3-M0 | + | + | ‒ |
| H191 | M | 39 | Tonsil | T2-N2b-M0 | + | + | ‒ |
| H202 | M | 81 | Tongue base | T3-N3-M0 | + | + | + |
| H206 | M | 58 | Tonsil | T2-N2b-M0 | + | + | + |

**Additional file 1: Table SI. Patient characteristics**

^*^ “H” indicate OPSCC patients included in the P07-112 head and neck cancer study.

^$^ Immune response status of OPSCC tumors was determined by analyzing cultured tumor infiltrating lymphocyte (TIL) batches for the presence of HPV16-specific T cells using a 5-day [3H]-thymidine-based proliferation assay and antigen-specific cytokine production assay as described and previously reported for most of these patients [16, 18]

Abbreviations: F: female; M: male; pTNM: pathological Tumor, lymph Nodes, Metastasis;
