# Supplementary material for: Tbet-positive regulatory T cells accumulate in oropharyngeal cancers with ongoing tumor-specific type 1 T cell responses
Source: J Immunother Cancer. 2019 Jan 18;7:14. doi: 10.1186/s40425-019-0497-0 (PMC6339415; doi:10.1186/s40425-019-0497-0)
Supplement: Supplementary file 2 — Figure S1. HPV16 E6/E7 specific T cell reactivity testing. Figure S2. Conventional (Tbet–) and Tbet+Foxp3+ Tregs can be found in tumor and stroma of OPSCC samples. Figure S3. The correlation between the number of CD8–Foxp3 + Tbet+ Tregs and CD8–Foxp3–Tbet+ (CD4) T cells and CD8 + Foxp3–Tbet+ T cells is retained in IR- and IR+ OPSCC tumors. Figure S4. Gating strategy for Treg subpopulations. Figure S5. TbethiFoxp3+ Tregs express higher levels of helios, CTLA4 and Ki67. Figure S6. The levels of tumor-infiltrating Foxp3hiTbethi Tregs correlated with the levels of infiltrating CD4 + Tbet+ and CD8 + Tbet+ cells, as well as with levels of highly activated infiltrating CD4+ and CD8+ T cells. Figure S7. Sorting of Tregs. (PPTX3379 kb) [file 40425_2019_497_MOESM2_ESM.pptx]

## Slide 1
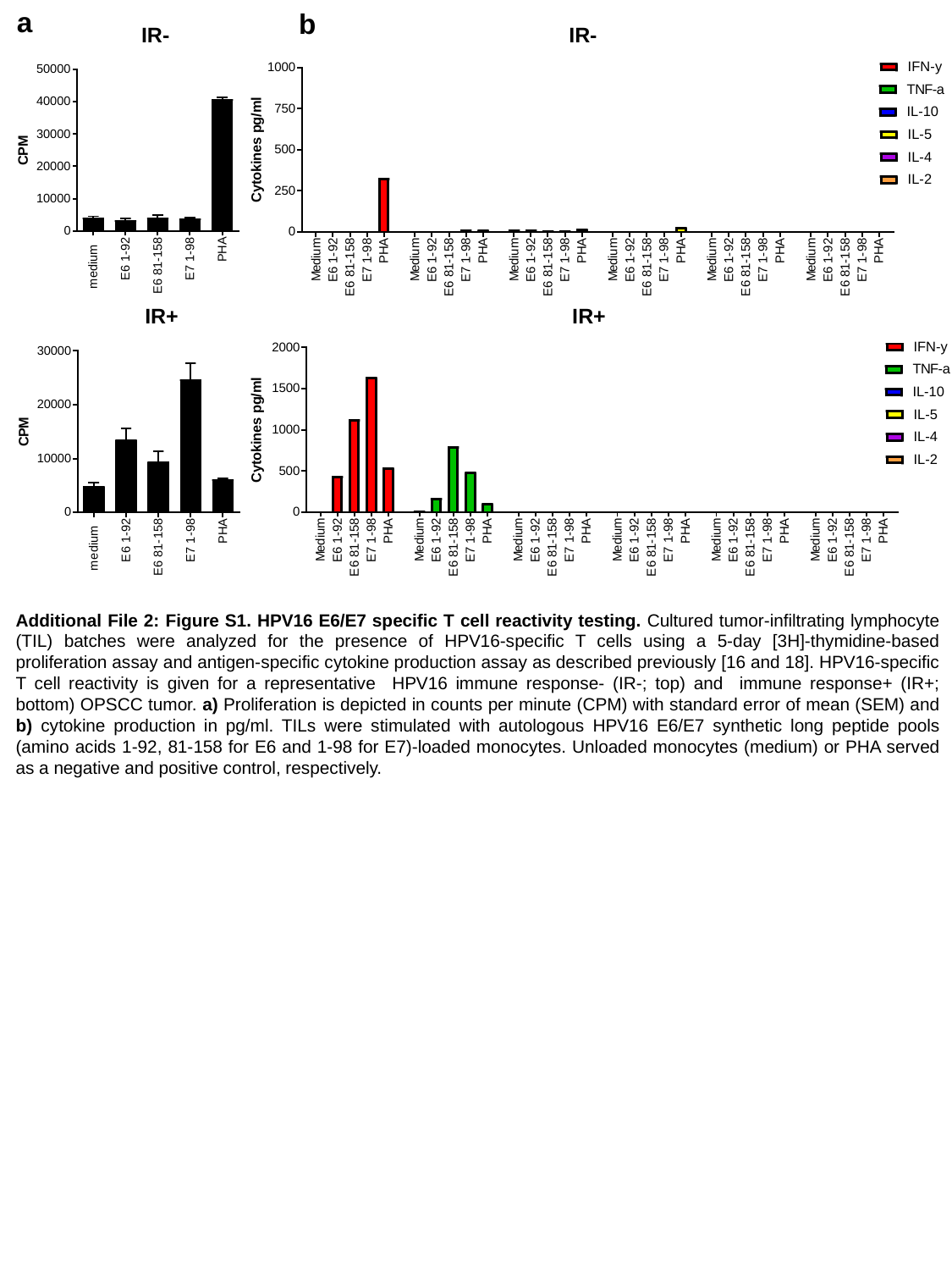

b
a
IR-
IR-
IR+
IR+
Additional File 2: Figure S1. HPV16 E6/E7 specific T cell reactivity testing. Cultured tumor-infiltrating lymphocyte (TIL) batches were analyzed for the presence of HPV16-specific T cells using a 5-day [3H]-thymidine-based proliferation assay and antigen-specific cytokine production assay as described previously [16 and 18]. HPV16-specific T cell reactivity is given for a representative HPV16 immune response- (IR-; top) and immune response+ (IR+; bottom) OPSCC tumor. a) Proliferation is depicted in counts per minute (CPM) with standard error of mean (SEM) and b) cytokine production in pg/ml. TILs were stimulated with autologous HPV16 E6/E7 synthetic long peptide pools (amino acids 1-92, 81-158 for E6 and 1-98 for E7)-loaded monocytes. Unloaded monocytes (medium) or PHA served as a negative and positive control, respectively.

## Slide 2
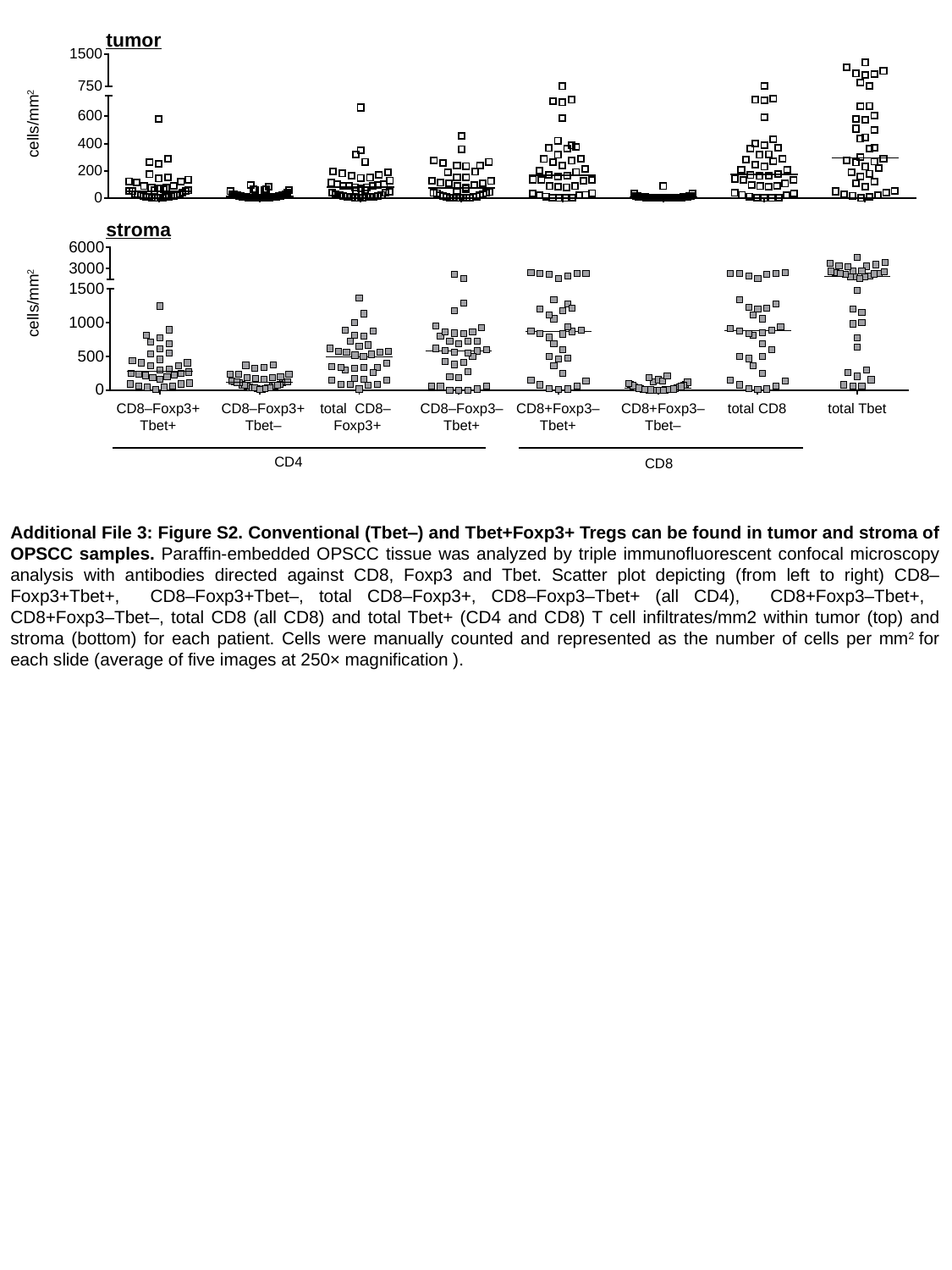

tumor
cells/mm2
stroma
cells/mm2
CD8‒Foxp3+
Tbet+
CD8‒Foxp3+
Tbet‒
total CD8‒
Foxp3+
CD8‒Foxp3‒
Tbet+
CD8+Foxp3‒
Tbet+
CD8+Foxp3‒
Tbet‒
total CD8
total Tbet
CD4
CD8
Additional File 3: Figure S2. Conventional (Tbet‒) and Tbet+Foxp3+ Tregs can be found in tumor and stroma of OPSCC samples. Paraffin-embedded OPSCC tissue was analyzed by triple immunofluorescent confocal microscopy analysis with antibodies directed against CD8, Foxp3 and Tbet. Scatter plot depicting (from left to right) CD8‒Foxp3+Tbet+, CD8‒Foxp3+Tbet‒, total CD8‒Foxp3+, CD8‒Foxp3‒Tbet+ (all CD4), CD8+Foxp3‒Tbet+, CD8+Foxp3‒Tbet‒, total CD8 (all CD8) and total Tbet+ (CD4 and CD8) T cell infiltrates/mm2 within tumor (top) and stroma (bottom) for each patient. Cells were manually counted and represented as the number of cells per mm2 for each slide (average of five images at 250× magnification ).

## Slide 3
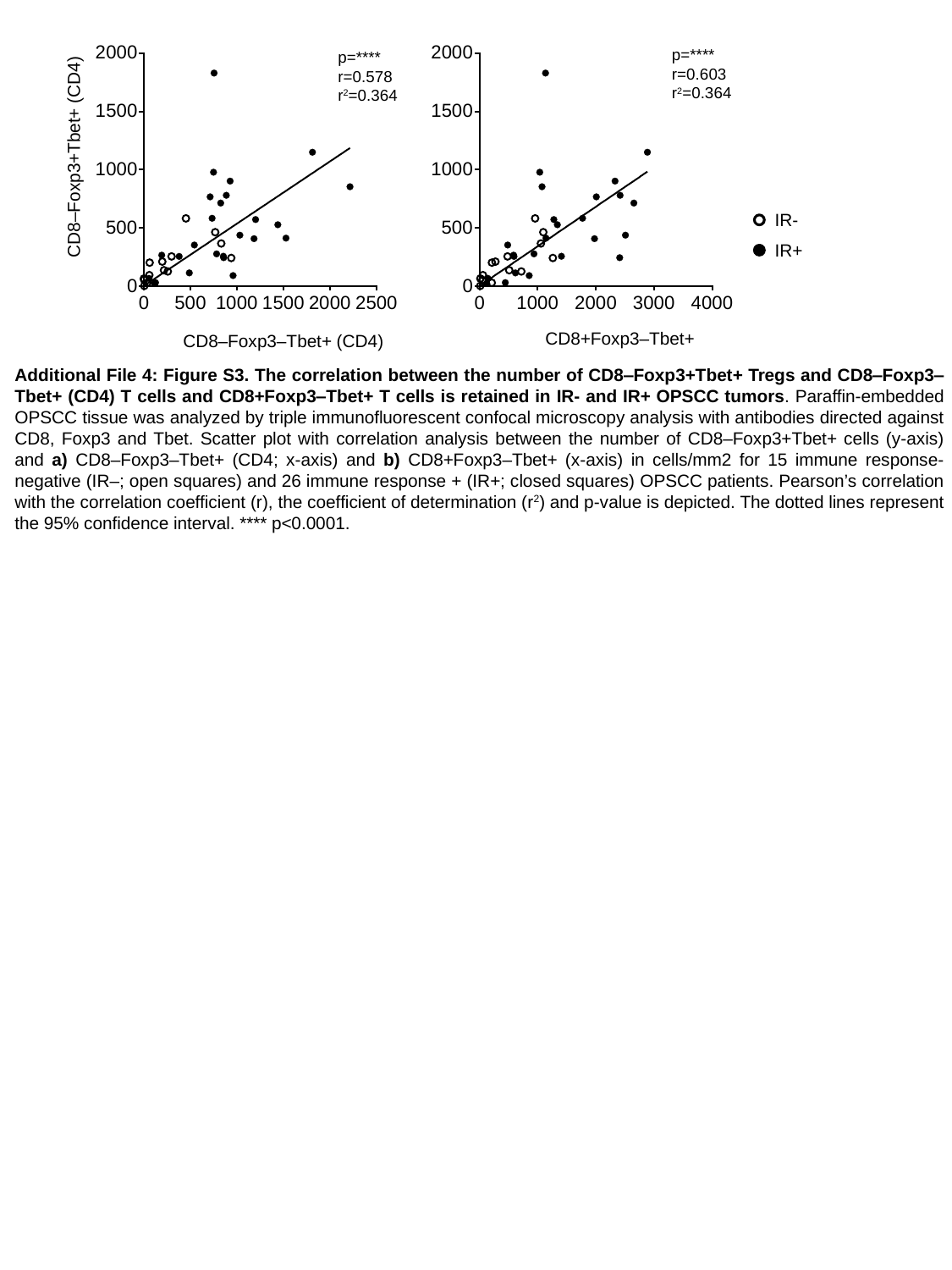

p=****
r=0.603
r2=0.364
p=****
r=0.578
r2=0.364
CD8‒Foxp3+Tbet+ (CD4)
IR-
IR+
CD8+Foxp3‒Tbet+
CD8‒Foxp3‒Tbet+ (CD4)
Additional File 4: Figure S3. The correlation between the number of CD8‒Foxp3+Tbet+ Tregs and CD8‒Foxp3‒Tbet+ (CD4) T cells and CD8+Foxp3‒Tbet+ T cells is retained in IR- and IR+ OPSCC tumors. Paraffin-embedded OPSCC tissue was analyzed by triple immunofluorescent confocal microscopy analysis with antibodies directed against CD8, Foxp3 and Tbet. Scatter plot with correlation analysis between the number of CD8‒Foxp3+Tbet+ cells (y-axis) and a) CD8‒Foxp3‒Tbet+ (CD4; x-axis) and b) CD8+Foxp3‒Tbet+ (x-axis) in cells/mm2 for 15 immune response-negative (IR‒; open squares) and 26 immune response + (IR+; closed squares) OPSCC patients. Pearson’s correlation with the correlation coefficient (r), the coefficient of determination (r2) and p-value is depicted. The dotted lines represent the 95% confidence interval. **** p<0.0001.

## Slide 4
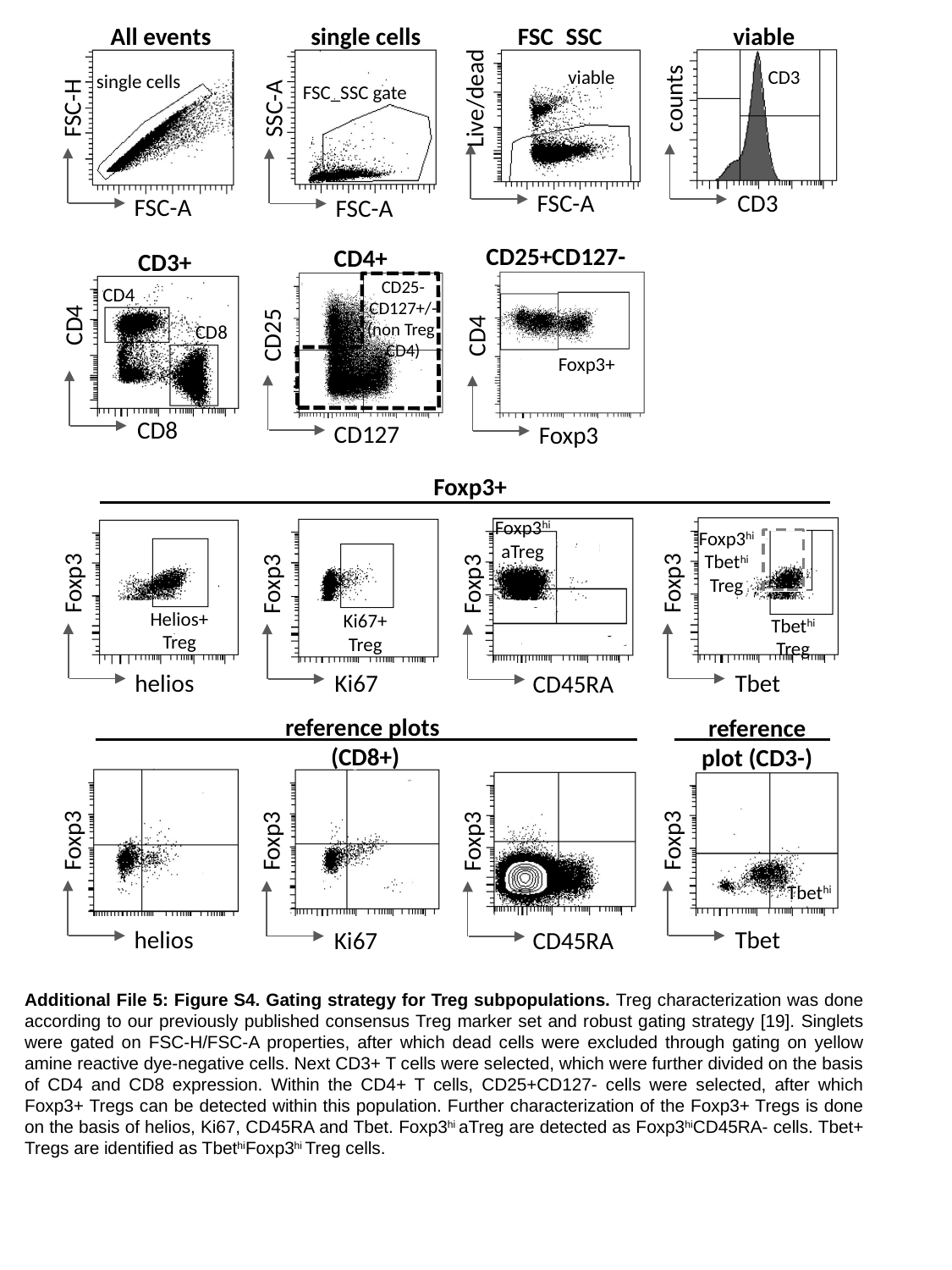

All events
single cells
FSC_SSC
viable
Live/dead
FSC-A
viable
CD3
counts
CD3
single cells
FSC-H
Tbet-
Tbet+
FSC-A
SSC-A
FSC-A
FSC_SSC gate
CD25+CD127-
CD4+
CD3+
CD25-
CD127+/-
(non Treg
CD4)
CD4
CD4
CD8
CD25
CD127
CD4
Foxp3
CD8
Foxp3+
Foxp3+
Foxp3hi
aTreg
Foxp3hi
Tbethi
Treg
Foxp3
Tbet
Foxp3
helios
Foxp3
Ki67
Foxp3
CD45RA
Helios+
Treg
Ki67+
Treg
Tbethi
Treg
reference plots
(CD8+)
reference plot (CD3-)
Foxp3
Tbet
Foxp3
helios
Foxp3
Ki67
Foxp3
CD45RA
Tbethi
Additional File 5: Figure S4. Gating strategy for Treg subpopulations. Treg characterization was done according to our previously published consensus Treg marker set and robust gating strategy [19]. Singlets were gated on FSC-H/FSC-A properties, after which dead cells were excluded through gating on yellow amine reactive dye-negative cells. Next CD3+ T cells were selected, which were further divided on the basis of CD4 and CD8 expression. Within the CD4+ T cells, CD25+CD127- cells were selected, after which Foxp3+ Tregs can be detected within this population. Further characterization of the Foxp3+ Tregs is done on the basis of helios, Ki67, CD45RA and Tbet. Foxp3hi aTreg are detected as Foxp3hiCD45RA- cells. Tbet+ Tregs are identified as TbethiFoxp3hi Treg cells.

## Slide 5
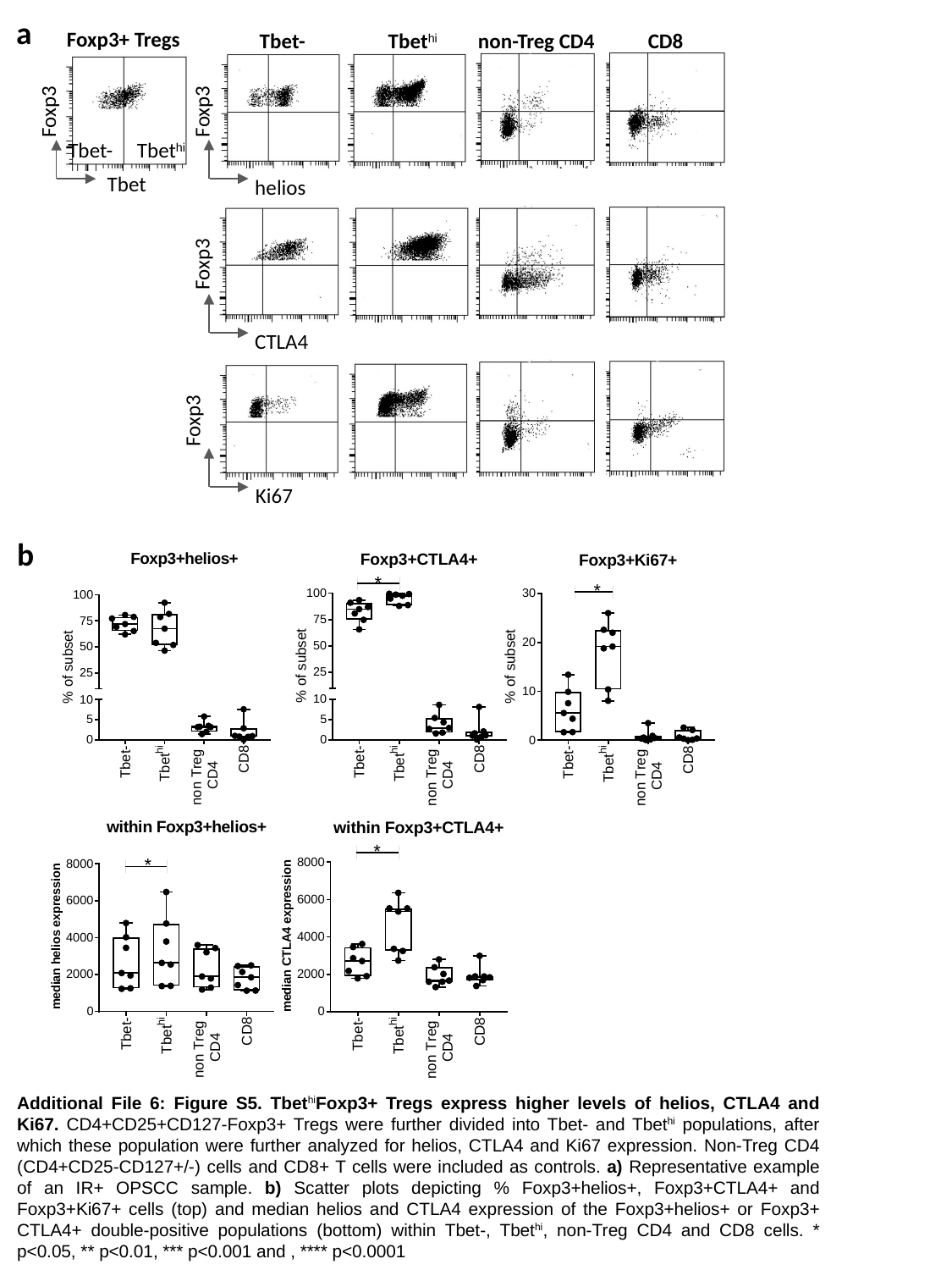

a
Foxp3+ Tregs
Tbet-
Tbethi
non-Treg CD4
CD8
Foxp3
helios
Foxp3
Tbet
Tbet-
Tbethi
Foxp3
CTLA4
Foxp3
Ki67
b
Additional File 6: Figure S5. TbethiFoxp3+ Tregs express higher levels of helios, CTLA4 and Ki67. CD4+CD25+CD127-Foxp3+ Tregs were further divided into Tbet- and Tbethi populations, after which these population were further analyzed for helios, CTLA4 and Ki67 expression. Non-Treg CD4 (CD4+CD25-CD127+/-) cells and CD8+ T cells were included as controls. a) Representative example of an IR+ OPSCC sample. b) Scatter plots depicting % Foxp3+helios+, Foxp3+CTLA4+ and Foxp3+Ki67+ cells (top) and median helios and CTLA4 expression of the Foxp3+helios+ or Foxp3+ CTLA4+ double-positive populations (bottom) within Tbet-, Tbethi, non-Treg CD4 and CD8 cells. * p<0.05, ** p<0.01, *** p<0.001 and , **** p<0.0001

## Slide 6
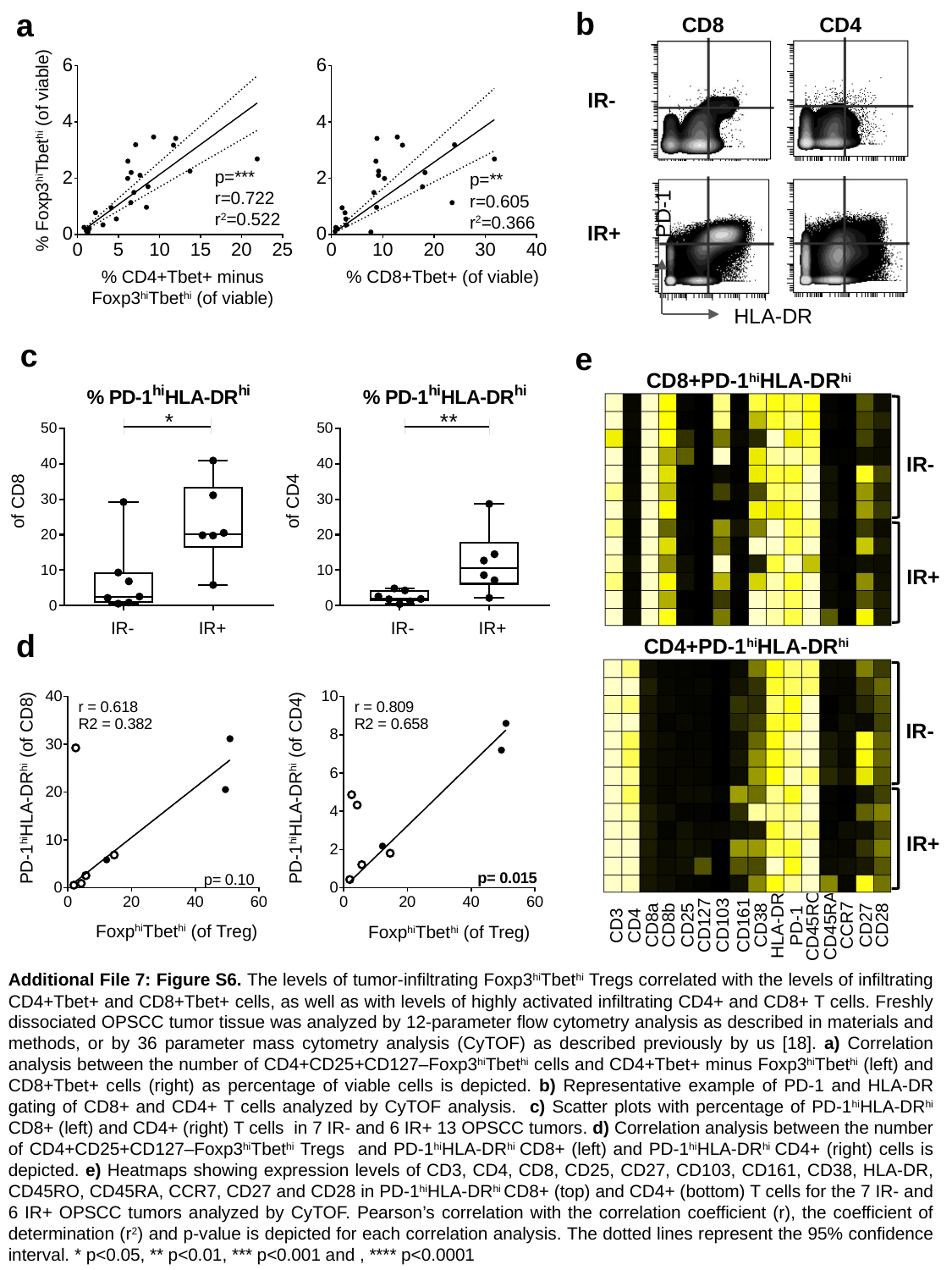

b
a
CD8
CD4
IR-
IR+
% Foxp3hiTbethi (of viable)
p=***
r=0.722
r2=0.522
p=**
r=0.605
r2=0.366
PD-1
HLA-DR
% CD4+Tbet+ minus Foxp3hiTbethi (of viable)
% CD8+Tbet+ (of viable)
c
e
CD8+PD-1hiHLA-DRhi
IR-
IR+
CD4+PD-1hiHLA-DRhi
IR-
IR+
CD3
CD4
CD8a
CD8b
CD25
CD127
CD103
CD161
CD38
HLA-DR
PD-1
CD45RO
CD45RA
CCR7
CD27
CD28
of CD8
of CD4
IR-
IR+
IR-
IR+
d
PD-1hiHLA-DRhi (of CD4)
PD-1hiHLA-DRhi (of CD8)
FoxphiTbethi (of Treg)
FoxphiTbethi (of Treg)
Additional File 7: Figure S6. The levels of tumor-infiltrating Foxp3hiTbethi Tregs correlated with the levels of infiltrating CD4+Tbet+ and CD8+Tbet+ cells, as well as with levels of highly activated infiltrating CD4+ and CD8+ T cells. Freshly dissociated OPSCC tumor tissue was analyzed by 12-parameter flow cytometry analysis as described in materials and methods, or by 36 parameter mass cytometry analysis (CyTOF) as described previously by us [18]. a) Correlation analysis between the number of CD4+CD25+CD127‒Foxp3hiTbethi cells and CD4+Tbet+ minus Foxp3hiTbethi (left) and CD8+Tbet+ cells (right) as percentage of viable cells is depicted. b) Representative example of PD-1 and HLA-DR gating of CD8+ and CD4+ T cells analyzed by CyTOF analysis. c) Scatter plots with percentage of PD-1hiHLA-DRhi CD8+ (left) and CD4+ (right) T cells in 7 IR- and 6 IR+ 13 OPSCC tumors. d) Correlation analysis between the number of CD4+CD25+CD127‒Foxp3hiTbethi Tregs and PD-1hiHLA-DRhi CD8+ (left) and PD-1hiHLA-DRhi CD4+ (right) cells is depicted. e) Heatmaps showing expression levels of CD3, CD4, CD8, CD25, CD27, CD103, CD161, CD38, HLA-DR, CD45RO, CD45RA, CCR7, CD27 and CD28 in PD-1hiHLA-DRhi CD8+ (top) and CD4+ (bottom) T cells for the 7 IR- and 6 IR+ OPSCC tumors analyzed by CyTOF. Pearson’s correlation with the correlation coefficient (r), the coefficient of determination (r2) and p-value is depicted for each correlation analysis. The dotted lines represent the 95% confidence interval. * p<0.05, ** p<0.01, *** p<0.001 and , **** p<0.0001

## Slide 7
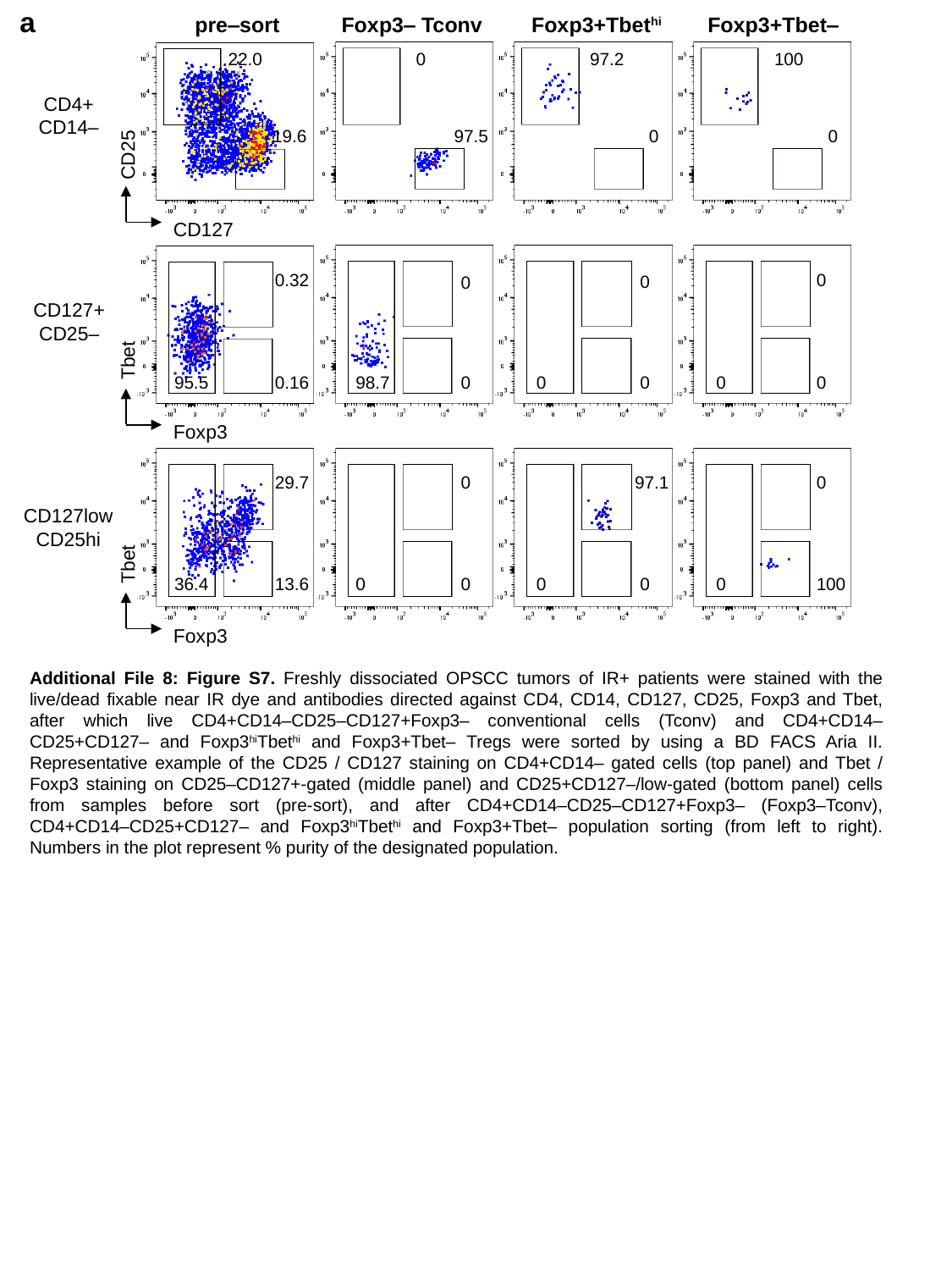

a
pre‒sort
Foxp3‒ Tconv
Foxp3+Tbethi
Foxp3+Tbet‒
22.0
0
97.2
100
CD4+
CD14‒
19.6
97.5
0
0
CD25
CD127
0.32
0
0
0
CD127+
CD25‒
Tbet
Foxp3
95.5
0.16
98.7
0
0
0
0
0
29.7
0
97.1
0
CD127low
CD25hi
Tbet
13.6
Foxp3
36.4
0
0
0
0
0
100
Additional File 8: Figure S7. Freshly dissociated OPSCC tumors of IR+ patients were stained with the live/dead fixable near IR dye and antibodies directed against CD4, CD14, CD127, CD25, Foxp3 and Tbet, after which live CD4+CD14‒CD25‒CD127+Foxp3‒ conventional cells (Tconv) and CD4+CD14‒CD25+CD127‒ and Foxp3hiTbethi and Foxp3+Tbet‒ Tregs were sorted by using a BD FACS Aria II. Representative example of the CD25 / CD127 staining on CD4+CD14‒ gated cells (top panel) and Tbet / Foxp3 staining on CD25‒CD127+-gated (middle panel) and CD25+CD127‒/low-gated (bottom panel) cells from samples before sort (pre-sort), and after CD4+CD14‒CD25‒CD127+Foxp3‒ (Foxp3‒Tconv), CD4+CD14‒CD25+CD127‒ and Foxp3hiTbethi and Foxp3+Tbet‒ population sorting (from left to right). Numbers in the plot represent % purity of the designated population.
